# C10X polymorphism in the *CARD8* gene is associated with bacteraemia

**DOI:** 10.1002/iid3.14

**Published:** 2013-11-05

**Authors:** Berhane Asfaw Idosa, Berolla Sahdo, Ermias Balcha, Anne Kelly, Bo Söderquist, Eva Särndahl

**Affiliations:** 1 Department of Clinical Medicine, School of Health and Medical Sciences, Örebro UniversitySE-701 82, Örebro, Sweden; 2 Department of Laboratory Medicine, Clinical Microbiology, Örebro University HospitalSE-701 85, Örebro, Sweden; 3 Faculty of Medicine and Health, Örebro UniversitySE-701 82, Örebro, Sweden

**Keywords:** Bacteraemia, blood culture, gene variants, infection, inflammasomes, inflammation, innate immunity, leukocytes, polymorphisms, sepsis

## Abstract

The NLRP3 inflammasome is an intracellular multi-protein complex that triggers caspase-1 mediated maturation of interleukin-1β (IL-1β); one of the most potent mediators of inflammation and a major cytokine produced during severe infections, like sepsis. However, the excessive cytokine levels seem to stage for tissue injury and organ failure, and high levels of IL-1β correlates with severity and mortality of sepsis. Instead, recent data suggest caspase-1 to function as a guardian against severe infections. CARD8 has been implied to regulate the synthesis of IL-1β via interaction to caspase-1. In recent years, polymorphism of CARD8 (C10X) per se or in combination with NLRP3 (Q705K) has been implicated with increased risk of inflammation. The aim was to investigate the correlation of these polymorphisms with severe blood stream infection. Human DNA was extracted from blood culture bottles that were found to be positive for microbial growth (i.e. patients with bacteraemia). Polymorphisms Q705K in the NLRP3 gene and C10X in the CARD8 gene were genotyped using TaqMan genotyping assay. The results were compared to healthy controls and to samples from patients with negative cultures. The polymorphism C10X was significantly over-represented among patients with bacteraemia as compared to healthy controls, whereas patients with negative blood culture were not associated with a higher prevalence. No association was observed with polymorphism Q705K of NLRP3 in either group of patients. Patients carrying polymorphism C10X in the CARD8 gene are at increased risk of developing bacteraemia and severe inflammation.

## Introduction

The innate immune system has evolved as a system that control microbial infections. Upon sensing the presence of pathogens, host innate immune cells initiate a broad spectrum of defence mechanisms that results in the development of inflammation and host resistance to infection. A key component of cytosolic surveillance is the NLRP3 inflammasome (reviewed in: [1]). By sensing a variety of microbial components, as well as endogenous and exogenous danger molecules, NLRP3 forms a multi-protein inflammasome complex that controls the activation of the proteolytic enzyme caspase-1. Caspase-1 in turn regulates the maturation of the proinflammatory cytokines: interleukin (IL)-1β and IL-18. IL-1β is one of the most potent mediators of inflammation, for example causing leukocytosis and fever [2]. IL-1β is also one of the important cytokines produced during severe infection, such as invasive infections. Despite this proinflammatory involvement, it is well documented that the innate immune system in septic patients is suppressed and unable to clear pathogens. The excessive cytokine levels seem rather to stage for tissue injury and organ failure [3], and high levels of IL-1β correlate with severity of disease and mortality [4]. Recent data indicate instead a role for caspase-1 in controlling severe infections [5–7]. CARD8 (also known as TUCAN/CARDINAL) has been found to be involved in NFκB-mediated suppression of the immune response and inflammatory activities [8]. A role in inflammasome-mediated processes has been proposed as CARD8 has been found to regulate IL-1β secretion [9] and cell death [10,11], probably by a direct physical interaction to caspase-1.

Variations in genes encoding the NLRP3 inflammasome are associated with auto-inflammatory diseases, and Q705K in *NLRP3* and C10X in *CARD8* are two polymorphisms that, per se or combined, have been associated with increased risk and severity of chronic inflammation [12–19]. The Q705K polymorphism renders NLRP3 into a gain-of-function phenotype [20], which results in a lower threshold for activation, whereas the C10X polymorphism results in a truncated non-functional protein and thus the loss of CARD8-mediated inhibition of caspase-1 [11]; that is both polymorphisms create a more susceptible inflammasome, which could be detrimental if leading to an inappropriate immune response. Actually, this could display a higher incidence or severity of inflammation in the setting of severe infections. In the present study, severely ill patients with suspected bacteraemia were investigated to find out if genetic variations of the NLRP3 inflammasome influence susceptibility to develop blood stream infection. The polymorphism C10X in the *CARD8* gene was found to be significantly over-represented among patients with bacteraemia as compared to healthy controls, whereas patients with negative blood culture were not associated with a higher prevalence.

## Materials and Methods

### Study subjects

Blood from a total of 100 positive and 100 negative blood culture bottles (BD BACTEC plus aerobic and anaerobic bottles; Becton, Dickinson and company, Franklin Lakes, NJ, USA) were collected consecutively during 2 months (January–February, 2012) by the accredited Department of Laboratory Medicine, Clinical Microbiology, Örebro University Hospital, Sweden. Each sample originated from a distinct patient. The infectious agents (bacterium and fungi) were isolated and identified according to routine laboratory procedures. For each distinct patient, four separate blood samples, two anaerobic and two aerobic, were cultured. If no indication of growth was obtained within 7 days, the samples were considered as negative. Coagulase-negative staphylococci (CoNS) were regarded as significant if growth were detected in two or more of the four separate blood culture bottles.

From the blood, human DNA was successfully extracted from 70 patients (60% male and 40% female; mean age: 69 (range: 0–99) years) with bacteraemia (positive) and from 76 patients (54% male and 46% female; mean age. 61 (range: 1–96) years) with no growth of microbes (negative), whereas human DNA was undetectable in the rest of the samples and these samples were thereby excluded from the study. One-thousand-three healthy individuals, previously analyzed [21], were used as controls (63% male, 37% female; mean age: 42 (range: 19–67) years). The variant allele frequencies of Q705K (rs35829419) in *NLRP3* and C10X (rs2043211) in *CARD8* genes in the control group were 7.2% and 32.9%, respectively [21], which is in agreement with previous studies [12,17] and the National Center for Biotechnology Information (NCBI) reference assembly on European population. Both polymorphisms were found to be in Hardy–Weinberg equilibrium.

### DNA extraction

DNA was isolated directly from blood culture bottles by pre-chemical lysis treatment and automated extraction method using Roche MagNA pure instrument (Roche Diagnostics GmbH, Mannheim, Germany) in accordance with the manufacturer's instructions. 2 mL aliquots of broth from blood culture bottles were centrifuged at 140*g* for 10 min and 200 µL supernatant was removed for further processing. Prior to MagNA pure compact, the specimens were treated with 10 µL of mutanolysin (Roche Diagnostic GmbH) and incubated at 37°C for 10 min. 180 µL of bacterial lysis buffer (Roche Diagnostic GmbH) and 20 µL proteinase K (Roche Diagnostic GmbH) were added to the specimens. After brief vortexing, specimens were incubated for 10 min at 65°C and subsequently, subjected to 95°C boiling for 10 min. Treated samples (400 µL) were transferred to the MagNA Pure Compact instrument for automated DNA extraction using prefilled cartridge MagNA pure compact Nucleic Acid Isolation Kits I (Roche Diagnostic GmbH). DNA concentration was measured using a Thermo Scientific NanoDrop™ 1000 Spectrophotometer (Thermo Fisher Scientific Wilmington, DE, USA). DNA was stored at −20°C until being used for further analysis.

### Genotyping by real-time PCR

The genotyping was performed for the polymorphisms Q705K (rs35829419) and C10X (rs2043211) in the *NLRP3* and *CARD8* genes, respectively, using DNA samples extracted from blood culture bottles. The analysis was performed by a TaqMan® SNP genotyping assay with 7900HT Fast Real-Time PCR system (Applied Biosystems, Foster City, CA, USA) followed by allelic discrimination to evaluate the frequencies of the different alleles. A volume of 2 µL genomic DNA was amplified in a final 20 µL reaction volume containing 10 µL 2x TaqMan® Genotyping Master Mix (Applied Biosystems), 0.5 µL of 20x TaqMan® SNP Genotyping assays Pre-designed primers and probes. Regarding the TaqMan amplification cycles, a cut-off of <35 cycles was used, and for most samples in the present study, the cycles were below 30, which obviously must be considered as a low number of amplification cycles and should not result in any risk of false assignment.

### Ethical considerations

The study was conducted in accordance with the Declaration of Helsinki. The blood samples were anonymized, and the only information given was the year of birth and gender of the patients. Since only anonymized samples were delivered to the researchers; thereby preventing data to be traced back to a certain individual, and since the blood was withdrawn from blood culture bottles and not directly from the patients; thereby causing no extra harm to the patients, the study did not require ethical approval according to paragraph 4 of the Swedish law (2003:460) on Ethical Conduct in Human Research.

### Statistical analyses

Hardy–Weinberg equilibrium was calculated in accordance with standard procedures using chi-square analysis (*χ*^2^) [22]. The results were analyzed by SPSS 17.0 software package and Prism 5, GraphPad Software. *χ*^2^-test was performed to compare genotype and allele frequencies, or Fisher's exact test. Moreover, binary logistic regression was used to calculate Odds ratio (OR) at corresponding 95% confidence interval (95% CI). Two-sided statistical significance value, *P* < 0.05 were considered as significant.

## Results

### Genotype distribution and allele frequencies of Q705K in the NLRP3 gene and C10X in the CARD8 gene

The classification of the microorganisms found in patients with bacteraemia in the present study is detailed in Table1. *Propionibacterium sp*. and *Lactobacillus* are generally non-invasive bacteria, and thus regarded as non-pathogenic. Therefore, these two samples were excluded from the study. In 15 samples, the identified bacterium was coagulase-negative *Staphylococcus sp*. (CoNS). Out of these, eight samples were found to be positive for growth of CoNS in two or more out of the four blood culture bottles, run in parallel for each patient, and were thus considered as true bacteraemia cases and included in the study.

**Table:** Distribution of pathogens identified in blood culture bottles of seventy consecutive patients, including the patients included in the study

Pathogens	Positive samples (*n* = 70)	Patients included (*n* = 60)
Gram positive infection		
Coagulase-negative staphylococci^1^	15	8
*Staphylococcus aureus*	9	9
*Streptococcus pneumoniae*	10	10
*Enterococcus faecium*	1	1
*Propionibacterium sp*.	2	0
*Streptococcus pyogenes*	2	2
*Enterococcus faecalis*	3	3
*Lactobacillus sp*.	1	0
*Streptococcus agalactiae*	1	1
*Clostridium ramosum*	1	1
Gram negative infection		
*Escherichia coli*	15	15
*Klebsiella pneumonia*	4	4
*Enterobacter cloacae*	1	1
*Fusobacterium sp*.	1	1
*Proteus mirabilis*	1	1
*Klebsiella oxytoca*	1	1
*Haemophilus influenzae*	1	1
*Bacteroides vulgatus*	1	1
Fungal		
*Candida albicans*^2^	1	0

1 At least two out of four cultures from a patient needed to signal positive for growth of Coagulase-negative *Staphylococcus* to be included in the study.

2 Fungi (*C. albicans*) was excluded from the analysis since the infection occurred only in one patient.

The prevalence of C10X polymorphism in the *CARD8* gene was significantly higher in patients with bacteraemia compared to healthy controls (allele frequencies: 50.0% vs. 32.9%, *P* = 0.0001; Table2), but also compared to patients with negative blood cultures (allele frequencies: 50.0% vs. 32.9%, *P* = 0.004; Table2). Patients with bacteraemia were to a significantly greater extent heterozygote carriers of C10X in the *CARD8* gene (66.6%) than non-bacteraemia patients (55.3%; *P* = 0.01) or healthy controls (46.1%; *P* < 0.0001), and this difference was also evident when comparing the carriers of homozygote polymorphism of C10X in the *CARD8* gene (Table2). Patients with bacteraemia displayed no significant difference in genotype distribution and allele frequency of Q705K in the *NLRP3* gene compared to healthy individuals as well as to patients with negative blood cultures (Table3). The polymorphisms were not found to be in Hardy–Weinberg equilibrium (*P* < 0.05, *χ*^2^) in neither the bacteraemia nor the non-bacteraemia group.

**Table:** Genotype and allele frequencies (%) in patients with bacteraemia (*n* = 60), patients with negative blood cultures (*n* = 76; non-bacteraemic samples) and healthy controls (*n* = 1003) for C10X (rs2043211) polymorphism in the *CARD8* gene

	Bacteraemic samples (%), *n* = 60	Non-bacteraemic samples (%), *n* = 76	OR (95% CI)^a^	*P-*value^a^	Healthy controls (%), *n* = 1003	OR (95% CI)^b^	*P-*value^b^
Genotype frequencies – *CARD8*							
CC	10 (16.7)	30 (39.5)	1		442 (44.1)	1	
CX	40 (66.6)	42 (55.3)	2.8 (1.2–6.5)	0.01	462 (46.1)	3.8 (1.8–7.7)	<0.0001
XX	10 (16.7)	4 (5.3)	7.5 (1.9–29.2)	0.004	99 (9.9)	4.4 (1.8–11.0)	0.001

	Bacteraemic samples (%), *n* = 120	Non-bacteraemic samples (%), *n* = 152	OR (95% CI)^a^	*P-*value^a^	Healthy controls (%), *n* = 2006	OR (95% CI)^b^	*P-*value^b^
Allele frequencies – *CARD8*							
C	60 (50.0)	102 (67.1)	1		1346 (67.1)	1	
X	60 (50.0)	50 (32.9)	2.0 (1.2–3.3)	0.004	660 (32.9)	2.0 (1.4–2.9)	0.0001

OR, odds ratio; CI, confidence interval.

a Association between bacteraemic and non-bacteraemic samples.

b Association between bacteraemic samples and healthy controls.

**Table:** Genotype and allele frequencies (%) in patients with bacteraemia (*n* = 60), patients with negative blood cultures (*n* = 76; non-bacteraemic samples) and healthy controls (*n* = 1003) for Q705K (rs35829419) polymorphism in the *NLRP3* gene

	Bacteraemic samples (%), *n* = 60	Non-bacteraemic samples (%), *n* = 76	OR (95% CI)^a^	*P-*value^a^	Healthy controls (%), *n* = 1003	OR (95% CI)^b^	*P-*value^b^
Genotype frequencies – *NLRP3*							
QQ	54 (90.0)	68 (89.5)	1		862 (85.9)	1	
QK	4 (6.7)	6 (7.9)	0.8 (0.2–3.1)	0.79	138 (13.8)	0.5 (0.2–1.2)	0.14
KK	2 (3.3)	2 (2.6)	1.3 (0.2–9.2)	0.72	3 (0.3)	10.6 (1.7–65)	0.01

	Bacteraemic samples (%), *n* = 120	Non-bacteraemic samples (%), *n* = 152	OR (95% CI)^a^	*P-*value^a^	Healthy controls (%), *n* = 2006	OR (95% CI)^b^	*P-*value^b^
Allele frequencies – *NLRP3*							
Q	112 (93.3)	142 (93.4)	1		1862 (92.8)	1	
K	8 (6.7)	10 (6.6)	1.0 (0.4–2.7)	0.97	144 (7.2)	0.9 (0.4–1.9)	0.83

OR, odds ratio; CI, confidence interval.

a Association between bacteraemic and non-bacteraemic samples.

b Association between bacteraemic samples and Healthy controls.

If CoNS samples that were regarded as positive, were excluded, the prevalence of C10X polymorphism in the *CARD8* gene was even more significant in patients with bacteraemia compared to healthy controls (allele frequencies: 51.0%, *P* < 0.0001; genotype: 67.3, *P* < 0.0001 (heterozygote) and 17.3, *P* = 0.001 (homozygote variant)).

### Combined genotype frequencies of Q705K in NLRP3 and C10X in CARD8 genes

The frequency of a combined heterozygous genotype (Q705K for the *NLRP3* gene plus C10X for the *CARD8*) in the healthy controls is 6.4% (Table S1 [21]), implying that approximately five patients with bacteraemia would be to expect in our study. However, only few patients displayed a combined heterozygous genotype (patients with bacteraemia: 1; non-bacteraemia patients: 3; Table S1). Due to this small sample size, no comparison of the presence of combined polymorphisms of Q705K in *NLRP3* gene plus C10X in the *CARD8* gene could be made between patients with bacteraemia and non-bacteraemia patients, nor compared to healthy individuals. Of note, whereas over 35% of the healthy individuals, as well as of the non-bacteraemia patients, were carriers of combined wild-type *CARD8* + *NLRP3*, only 11.7% of the patients with bacteraemia had this genotype (Table S1). None of the groups (neither patients nor controls) held individuals carrying the combined homozygous variant genotype XX of C10X plus KK of Q705K.

### Genotype distribution and allele frequencies of C10X in the CARD8 gene in patients with Gram-positive and Gram-negative infection, respectively

Genotype frequency of the polymorphism C10X in *CARD8* gene was analyzed among patients with bacteraemia comparing prevalence of infection caused by Gram-positive and Gram-negative bacteria, respectively. Patients were susceptible to infections caused by Gram-positive (*P* = 0.013) as well as Gram-negative bacteria (*P* = 0.002) compared to healthy controls (Table4), with the exception that the few carriers (*n* = 5) of homozygote variant XX of *CARD8* were not associated with infections caused by Gram-positive bacteria (*P* = 0.052). In addition, the prevalence of C10X polymorphism in *CARD8* gene was significantly higher in patients with bacteraemia caused by either Gram-negative bacteria (*P* = 0.008), or Gram-positive bacteria (*P* = 0.041) compared to patients with negative culture (Table5).

**Table:** Comparison of genotype frequencies (%) of C10X polymorphism (*CARD8* gene) in bacteraemic patients infected with Gram-positive G^+^; *n* = 35) and Gram-negative (G^−^; *n* = 25) bacteria to healthy controls (*n* = 1003)

	G^+^ samples (%), *n* = 35	Healthy controls (%), *n* = 1003	OR (95% CI)^a^	*P-*value^a^	G^−^ samples (%), *n* = 25	OR (95% CI)^b^	*P-*value^b^
Genotype frequencies – *CARD8*							
CC	7 (20)	442 (44.1)	1		3 (12)	1	
CX	23 (65.7)	462 (46.1)	3.1 (1.3–7.4)	0.009	17 (68)	5.4 (1.6–18.6)	0.007
XX	5 (14.3)	99 (9.9)	3.2 (1.0–10.3)	0.052	5 (20)	7.4 (1.7–31.7)	0.007

	G^+^ samples (%), *n* = 70	Healthy controls (%), *n* = 2006	OR (95% CI)^a^	*P-*value^a^	G^−^ samples (%), *n* = 50	OR (95% CI)^b^	*P-*value^b^
Allele frequencies – *CARD8*							
C	37 (52.9)	1346 (67.1)	1		23 (46)	1	
X	33 (47.1)	660 (32.9)	1.8 (1.1–2.9)	0.013	27 (54)	2.4 (1.4–4.2)	0.002

OR, odds ratio; CI, confidence interval.

a Association between G^+^ samples (patients with Gram-positive bacteria infection) and Healthy controls.

b Association between G^−^ samples (patients with Gram-negative bacteria infection) and Healthy controls.

**Table:** Comparison of genotype frequencies (%) of C10X polymorphism (*CARD8* gene) in bacteraemic patients infected with Gram-positive (G^+^; *n* = 35) and Gram-negative (G^−^; *n* = 25) bacteria to patients with negative blood culture bottles (non-bacteraemic patients; *n* = 76)

	G^+^ samples (%), *n* = 35	Non-bacteraemic samples (%), *n* = 76	OR (95% CI)^a^	*P-*value^a^	G^−^ samples (%), *n* = 25	OR (95% CI)^b^	*P-*value^b^
Genotype frequencies – *CARD8*							
CC	7 (20)	30 (39.5)	1		3 (12)	1	
CX	23(65.7)	42 (55.3)	2.3 (0.9–6.2)	0.084	17 (68)	4.0(1.1–15.1)	0.037
XX	5(14.3)	4 (5.3)	5.4 (1.1–25.3)	0.034	5 (20)	12.5(2.1–73.5)	0.005

	G^+^ samples (%), *n* = 70	Non-bacteraemic samples (%), *n* = 152	OR (95% CI)^a^	*P-*value^a^	G^−^ samples (%), *n* = 50	OR (95% CI)^b^	*P-*value^b^
Allele frequencies – *CARD8*							
C	37 (52.9)	102 (67.1)	1		23 (46)	1	
X	33 (47.1)	50 (32.9)	1.8 (1.02–3.2)	0.041	27 (54)	2.4 (1.2–4.6)	0.008

OR, odds ratio; CI, confidence interval.

a Association between G^+^ samples (patients with Gram-positive bacteria infection) and non-bacteremic patients.

b Association between G^−^ samples (patients with Gram-negative bacteria infection) and non-bacteremic patients.

## Discussion

The present study was designed as a case-control study in order to find out if genetic variations of the NLRP3 inflammasome are associated with susceptibility to severe infectious disease. The presence of polymorphisms Q705K (rs35829419) in *NLRP3* and C10X (rs2043211) in *CARD8* genes were investigated in patients with bacteraemia and compared to severely ill patients without detected bacteraemia, as well as to healthy individuals. The polymorphism C10X in the *CARD8* gene was significantly more prevalent in patients with bacteraemia compared to healthy individuals (*P* < 0.0001). The finding that the C10X polymorphism was not in Hardy–Weinberg equilibrium in the bacteraemic patients (*P* = 0.0018, *χ*^2^) makes the genotype difference between patients with bacteraemia and healthy controls even more evident. The polymorphism C10X causes a nonsense allele; resulting in reduced expression of CARD8 [11,23], which in turn is suggested to result in a loss of its inhibitory effect on caspase-1, and in so doing, caspase-1 is more easy to trigger. Miller and co-worker [11] speculated that C10X-carriers thereby have a more robust inflammatory response, and thus, better survive under conditions with higher infectious-disease burden, but could, on the other hand, manifest a pronounced inflammation during the acute phase of a severe infection. Our data most strongly support this idea by showing higher prevalence of C10X polymorphism in patients with bacteraemia; in whom septic symptoms may arise as a rapid consequence of an excessive caspase-1 response. In fact, judging from our data, homozygote carrier ship results in a phenotype more susceptible to infectious disease compared to heterozygote carriers; indicating that healthy C10X individuals (especially homozygote C10X) have a higher risk of severe bacteraemia in the case of infectious disease. In support of our finding, blocking caspase-1 decreases inflammation and mortality in rat models of endotoxin-induced shock [24,25]. Also, caspase-1 deficient mice are sheltered from disease of excessive inflammation, such as bacterial-induced sepsis [26], and are markedly resistant to the lethal effects of endotoxin [27]. These data, taken together with our finding of a high prevalence of wild-type *CARD8* allele in the healthy population, suggest that a functional *CARD8*, which thus controls caspase-1 activity, might provide a better protection against excessive, uncontrolled inflammation. However, the present study does not provide a mechanistic understanding of how the genetic variant C10X acts to alter disease susceptibility. Beside its role in maturation of IL-1 family cytokines, caspase-1 is known to process a number of other proteins [28]; which of many are proteins known to regulate the immune response, as well as to induce pyroptosis; a cell death that influence bacterial clearance independent of IL-1β and IL-18 [29]. Contradictory to our findings, several studies have shown that caspase-1-deficient mice are more susceptible to a number of pathogens [30–32]. Therefore, future studies are needed to understand how C10X modulate the function of the inflammasome in regulating host resistance mechanisms and by that the susceptibility to develop an infection.

CoNS are bacteria that constitute a major part of the normal flora, thus regarded as commensals, and could therefore be considered as a false positive result due to contamination when recovered in blood culture bottles. Since the patients in this study were anonymized, it is not possible to find out whether they represent true bacteraemia cases (e.g. immune-compromised patients, patients with indwelling devices or foreign bodies) or contaminations. Therefore, the most obvious cases of contamination, that is growth of CoNS in one out of four blood culture bottles were excluded, whereas growth of CoNS in two or more bottles were considered as true bacteraemia cases and thus included in the study. Whether CoNS are to be included in the study or not could be argued but if all CoNS samples are withdrawn from the calculations, the prevalence of C10X polymorphism in the *CARD8* gene is even more significant in patients with bacteraemia compared to healthy controls; data that argue that including the CoNS does, if anything, impair our results.

Most interestingly, our data show that patients with negative blood culture, that is patients that exhibit severe diseases without detectable bacteraemia, displayed similar expression of C10X as healthy individuals. It has been found that mRNA levels of caspase-1 are affected in patients with microbe-induced septic shock during the first 24 h of disease compared to critically ill intensive care unit patients [4]. This means that the cause of disease (in this case: infectious vs. non-infectious) are of crucial importance to understand the inflammatory response behind the disease, and most certainly therefore also in the treatment of disease. A need to establish the aetiology is most evident in a condition like sepsis, which manifest with similar clinical features even if presented from an infectious or non-infectious background. By separating patients with bacteraemia from non-bacteraemia patients, our study showed a strong correlation between the prevalence of genetic variation of *CARD8* and blood stream infection. This finding is supported by data showing only a weak association (*P* = 0.050) between the C10X-genotype and systemic inflammatory response syndrome (SIRS) when infectious and non-infectious SIRS patients were grouped and analyzed together [11]. Even if some caution should be taken into account when interpreting our results since the C10X polymorphism was not in Hardy–Weinberg equilibrium in the non-bacteraemic patients (*P* = 0.03, *χ*^2^); our data highlights the importance of investigating distinct groups of patients of well-defined aetiologies. Also, these data seem to indicate a contribution of C10X not only to bacteraemia but also the general clinical presentation of sepsis independent of the presence of blood-born infections. In addition, we found that patients carrying C10X are sensitive to infections caused by both Gram-positive and Gram-negative bacteria. Further investigations are needed to find out if all bacteria are causative in this genetic setting.

The C10X-genotype has been found associated with chronic consequences on human health by resulting in severity of autoimmune and auto-inflammatory disease [18,33,34]; probably due to a long-term effect of excessive caspase-1 response. We and others have found the combined polymorphism of C10X in *CARD8* and Q705K in *NLRP3* to be associated with inflammatory diseases [12,13] and with susceptibility to and severity of rheumatoid arthritis and Crohn's disease [16,17,19]. In this pilot study, only few patients were combined carriers of Q705K + C10X, and, therefore, no analysis of an association of a combined heterozygote Q705K + C10X genotype in patients with bacteraemia could be made. Larger cohorts are needed to investigate whether or not bacteraemia is associated with the combined Q705K + C10X genotype.

In conclusion, the present study showed a strong correlation between C10X polymorphism of the *CARD8* gene and the presence of bacteraemia. The current study was not designed to investigating whether C10X also correlates with disease outcome, that is survival or death, or solely to susceptibility of severe infectious disease, and future studies are therefore warranted elucidating this important question. Also, forthcoming studies should by ex vivo experiments validate the association between the gene variant and clinical course, and elucidate the molecular and cellular mechanisms by which the genetic variant C10X alters disease susceptibility.

## SUPPORTING INFORMATION

**Table S1.** Combined genotype frequencies (%) of the polymorphism C10X (rs2043211) in the CARD8 gene and Q705K (rs35829419) in the NLRP3 gene in patients with bacteraemia (*n* = 60), patients with negative blood cultures (*n* = 76; non-bacteraemic samples) and healthy controls (*n* = 1003).
