# Supplementary material for: C10X polymorphism in the CARD8 gene is associated with bacteraemia
Source: Immun Inflamm Dis. 2013 Nov 5;2(1):13–20. doi: 10.1002/iid3.14 (PMC4220665; doi:10.1002/iid3.14)
Supplement: Supplementary file 1 — Table S1. Combined genotype frequencies (%) of the polymorphism C10X (rs2043211) in the CARD8 gene and Q705K (rs35829419) in the NLRP3 gene in patients with bacteraemia (n = 60), patients with negative blood cultures (n = 76; non-bacteraemic samples) and healthy controls (n = 1003). [file iid30002-0013-SD1.pdf]

Supplementary Table SI.

Combined genotype frequencies (%) of the polymorphism C10X (rs2043211) in the *CARD8* gene and Q705K (rs35829419) in the *NLRP3* gene in patients with bacteraemia (n=60), patients with negative blood cultures (n=76; non-bacteraemic samples) and healthy controls (n=1003).

	**Combined genotype frequencies - *CARD8/NLRP3***		
	**Bacteraemic samples** (%), n=60	**Non-Bacteraemic samples** (%), n=76	**Healthy controls** (%), n=1003
**CC/QQ**	7 (11.7)	27 (35.5)	374 (37.3)
**CC/QK**	3 (5)	3 (3.9)	66 (6.6)
**CC/KK**	0	0	2 (0.2)
**CX/QQ**	37 (61.7)	37 (48.7)	397 (39.6)
**CX/QK**	1 (1.7)	3 (3.9)	64 (6.4)
**CX/KK**	2 (3.3)	2 (2.6)	1 (0.1)
**XX/QQ**	10 (16.7)	4 (5.3)	91 (9.1)
**XX/QK**	0	0	8 (0.8)
**XX/KK**	0	0	0
